# Preferential distribution of nuclear MAPK signal in α/β core neurons during long-term memory consolidation in *Drosophila*

**DOI:** 10.1007/s13238-017-0404-8

**Published:** 2017-04-19

**Authors:** Wantong Hu, Xuchen Zhang, Lianzhang Wang, Zhong-Jian Liu, Yi Zhong, Qian Li

**Affiliations:** 1 Tsinghua-Peking Center for Life Sciences, MOE Key Laboratory of Protein Sciences, IDG/McGovern Institute for Brain Research, School of Life Sciences, Tsinghua University, Beijing, 100084 China; 2 Shenzhen Key Laboratory for Orchid Conservation and Utilization, The National Orchid Conservation Center of China and The Orchid Conservation & Research Center of Shenzhen, Shenzhen, 518114 China; 3 The Center for Biotechnology and Biomedicine, Graduate School at Shenzhen, Tsinghua University, Shenzhen, 518055 China

**Dear Editor,**

Neuronal signal relay from synapse to nucleus, which is evoked by behavioral training, plays a vital part in consolidation of protein synthesis-dependent long-term memory (LTM) from invertebrates to vertebrates (Kandel et al., 2014). Among different training-induced neuronal signals, activation of MAPK (mitogen-activated protein kinase) is extensively studied and widely believed to be essential and critical for LTM consolidation from invertebrates to vertebrates (Alberini and Kandel, 2015). Extensive studies contribute to two fundamental questions that how behavioral training activates synaptic signaling molecules and how nuclear signaling molecules initiate new transcription of genes (Alberini and Kandel, 2015). However, relatively slower progress has been made on how behavioral training-induced synaptic signals translocate into nucleus, which is a critical step to bridge the former two questions together. In a recent study, we found that DIM-7, an importin in *Drosophila*, plays a critical role in mediating nuclear translocation of pMAPK to initiate LTM consolidation (Li et al., 2016). In that study, we found that Kenyon cells (KCs), neurons of mushroom body (MB), are critical places for nuclear translocation of pMAPK signal in determining LTM consolidation. Since the MB, which is a center of associative memory in *Drosophila* (Davis, 2005), contains about 2,000 neurons (Aso et al., 2009), it is interesting and useful to know whether such pMAPK nuclear translocation occurs evenly in all these neurons or preferentially in a specific group of KCs. In the current study, we combined behavioral training paradigm with confocal imaging to address this question. What we found is that consolidation-related pMAPK nuclear translocation occurs preferentially in a small group of MB neurons (α/βc KCs), which are reported to be necessary and specific for LTM consolidation (Huang et al., 2012).

According to our previous study (Li et al., 2016), we found that LTM training (spaced training, four repeated training sessions with 15-min interval) significantly induces more nuclear translocation of pMAPK at a representative time point of consolidation (8-h after spaced training), compared with naive flies and flies subjected to non-LTM training (massed training, four repeated and consecutive training sessions). This data indicates that LTM training specifically causes pMAPK nuclear translocation in KCs. In the current work, by using the same method, we employ more specific Gal4 lines to study the distribution of such training-induced nuclear pMAPK signal in subgroups of KCs.

We first checked the distribution of nuclear pMAPK signal in three major classes of MB neurons (α/β, γ, and α’/β’) at 8-h after spaced training, a representative time point during LTM consolidation (Li et al., 2016). To distinguish these classes, we employed three specific Gal4 lines: c739, VT44966, VT57244. These lines were reported to be specific drivers of different MB drivers (Aso et al., 2009; Wu et al., 2013; Yang et al., 2016). By crossing these Gal4 lines with *UAS-mCD8*::*GFP*; *MB247-DsRed* flies, we confirmed their specific expression patterns in MB lobes (Fig. S1). Relative to all MB lobes labeled by DsRed signal (red color), c739-Gal4, VT44966-Gal4, and VT57244-Gal4 showed strong and specific expression respectively in α/β lobe, γ lobe, and α’/β’ lobe (See GFP signal, green color). These Gal4 tools allow us to detect pMAPK signal in each specific type of KCs during LTM consolidation. The data were shown in Fig. 1. We crossed these Gal4 lines with *UAS-nlsGFP* flies to label the nuclei of specific KCs (GFP signal, green color). All nuclei in MB were labeled by TO-PRO-3 (blue color), while pMAPK signals were detected by its specific antibody (red color). From the representative images, we could see a clearly preferential distribution of pMAPK in MB nuclei (Fig. 1A). In contrast to γ KCs (VT44966) and α’/β’ KCs (VT57244), nuclear translocation of pMAPK occurred more likely in nuclei of α/β KCs (c739). Then we analyzed all the imaging data by measuring the mean intensity of nuclear pMAPK relative to calyx (the dendritic area of MB) and by counting the number of nuclei with strong pMAPK signal in different types of KCs. As Figure 1B showed, pMAPK mean intensity in nuclei of α/β KCs (c739) were significantly higher than γ KCs (VT44966) and α’/β’ KCs (VT57244). Consistently, the number of nuclei with strong pMAPK signal in α/β KCs (c739) was also apparently more than other two types of KCs (Fig. 1C). Interestingly, there were more nuclei with strong pMAPK signal in γ KCs (VT44966) compared with α’/β’ KCs (VT57244) (Fig. 1C), despite that there were no significant differences of nuclear pMAPK mean intensity between these two types of KCs (Fig. 1B). It indicates that nuclear translocation of pMAPK signal of these two types of KCs is different to a certain extent. Together, these findings support that pMAPK nuclear translocation occurs differently in different classes of KCs during consolidation stage, and showed a preferential distribution in α/β KCs.

**Figure 1:**
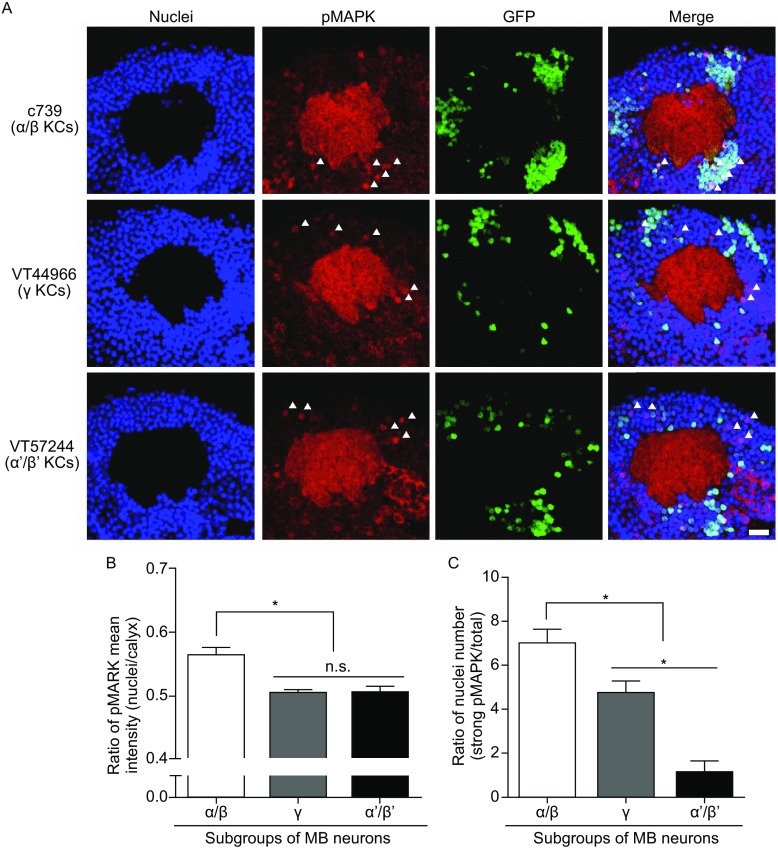
**Distribution of nuclear pMAPK signal in three major types of MB neurons during consolidation.** (A) Representative images of pMAPK signal in different types of MB neurons at 8-h after spaced training. Indicated Gal4 lines were crossed to flies with the genotype *UAS-nlsGFP* and detected by confocal imaging of whole adult central brain. Nuclei are indicated by blue color. pMAPK signal is displayed as red color. Gal4-drived expression of nlsGFP is shown as green color. Five representative nuclei with strong pMAPK signal are marked by white triangle. Scale bar is 10 μm. (B) Statistical analysis as reflected in mean intensity ratio of pMAPK signal (Gal4-labeled nuclei vs. calyx). α/β MB neurons showed significantly higher ratio of pMAPK mean intensity than γ and α’/β’ MB neurons. Bars, mean ± SEM (*n* = 6–7); *P < 0.05. (C) Statistical analysis as reflected in nuclear number ratio of strong pMAPK signal (number of Gal4-labeled nuclei with strong pMAPK vs. number of Gal4-labeled nuclei). Nuclear number ratio of strong pMAPK signal in α/β MB neurons is remarkably higher than in γ and α’/β’ MB neurons. Bars, mean ± SEM (*n* = 6–7); *P < 0.05

Next, we explored whether the distribution of nuclear pMAPK signal is still preferential in subgroups of α/β KCs. α/β KCs include about 1000 neurons and can be divided into at least three subgroups: α/β posterior (α/βp, ~75 neurons), α/β surface (α/βs, ~700 neurons), and α/β core (α/βc, ~200 neurons) (Aso et al., 2009). Among these three subgroups, α/βp KCs innervate only the accessary calyx and are dispensable for LTM (Huang et al., 2013; Perisse et al., 2013). In contrast, α/βs KCs and α/βc KCs innervate the main calyx and are closely linked with LTM (Huang et al., 2013; Huang et al., 2012; Perisse et al., 2013). Thus, our study focused on α/βs KCs and α/βc KCs. First, we used two specific Gal4 lines to distinguish these two subgroups: VT26665 for α/βs KCs and NP7175 for α/βc KCs. The expression pattern of these two Gal4 lines in MB lobes was shown in Fig. S2. Then with the help of these tools, we checked the distribution of pMAPK signal in nuclei at 8-h after spaced training as we did in Fig. 1. According to our data, both the nuclear pMAPK mean intensity and number of strong pMAPK nuclei in α/βc KCs (NP7175) were significantly higher than α/βs KCs (VT26665) (Fig. 2A–C). Since cell number of α/βc KCs is reported to be much fewer than α/βs KCs (Aso et al., 2009), it is possible that higher pMAPK signal observed in α/βc nuclei here might be caused by fewer cell number. To address this concern, we compared the distribution of all nuclei with strong pMAPK signal in both α/βc and α/βs neurons (Fig. 2D). Of note, the data showed that there was no significant difference found between these two groups of neurons, supporting that higher pMAPK signals observed in α/βc nuclei are not due to fewer cell number. Thus, pMAPK nuclear translocation also occurs differently in different subgroups of α/β KCs during consolidation stage, and showed a preferential distribution in α/βc KCs.

**Figure 2:**
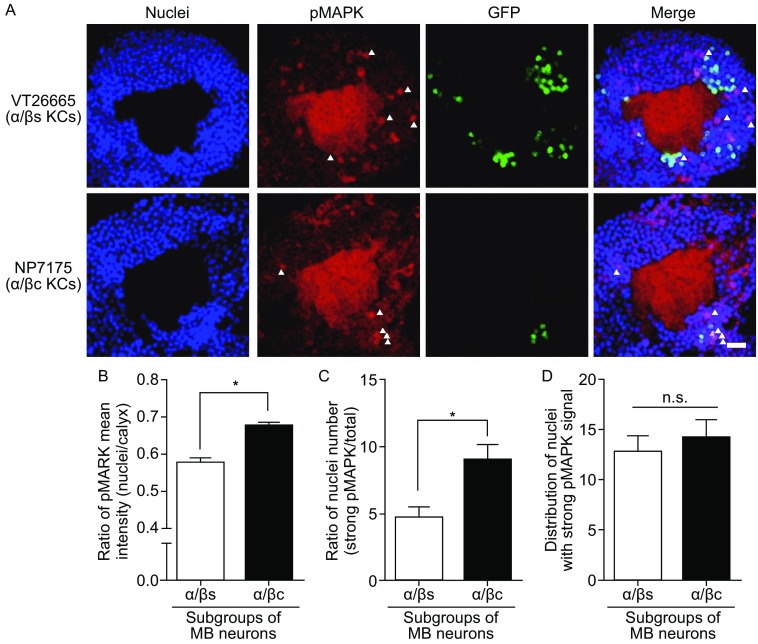
**Distribution of nuclear pMAPK signal in two subgroups of α/β KCs during consolidation.** (A) Representative images of pMAPK signal in different types of α/β KCs at 8-h after spaced training. Indicated Gal4 lines were crossed to flies with the genotype *UAS-nlsGFP* and detected by confocal imaging of whole adult central brain. Nuclei are labeled by blue color. pMAPK signal is displayed as red color. Gal4-drived expression of nlsGFP is shown as green color. Five representative nuclei with strong pMAPK signal are marked by white triangle. Scale bar is 10 μm. (B) Statistical analysis as reflected in mean intensity ratio of pMAPK signal (Gal4-labeled nuclei vs. calyx). α/βc KCs showed significantly higher ratio of pMAPK mean intensity than α/βs KCs. Bars, mean ± SEM (*n* = 6–7); *P < 0.05. (C) Statistical analysis as reflected in nuclear number ratio of strong pMAPK signal (number of Gal4-labeled nuclei with strong pMAPK vs. number of Gal4-labeled nuclei). Nuclear number ratio of strong pMAPK signal in α/βc KCs is remarkably higher than in α/βs KCs. Bars, mean ± SEM (*n* = 6–7); *P < 0.05. (D) Statistical analysis as reflected distribution of nuclei with strong pMAPK signal. No significant difference was found between α/βc and α/βs KCs. Bars, mean ± SEM (*n* = 6–7); *P < 0.05

According to our present findings, pMAPK nuclear translocation in α/β KCs is highly related to LTM consolidation in contrast to γ KCs and α’/β’ KCs. Consistently, α/β KCs are essential for aversive LTM at both neural circuit and molecular level (Huang et al., 2012; Isabel et al., 2004; Yu et al., 2006). In contrast, α’/β’ KCs are reported to be involved in early memory (Krashes et al., 2007; Wang et al., 2008). Interestingly, although neurotransmission of γ KCs is dispensable for LTM retrieval (Isabel et al., 2004), significant calcium trace in γ KCs is reported to be important for late LTM memory (Akalal et al., 2010). These two studies may help to explain our finding that there are more nuclei with strong pMAPK signal in γ KCs (VT44966) compared with α’/β’ KCs (VT57244) (Fig. 2C). To make sure the roles of γ, α’/β’, and α/β KCs in LTM consolidation, more studies should be needed.

Our finding strongly suggests that nuclear translocation of pMAPK in α/β core neurons is crucial for LTM consolidation. This suggestion is well supported by two previous studies in *Drosophila*. First, blocking the outputs of α/β core neurons during consolidation stage but not retrieval stage specifically impairs 24-h aversive LTM (Huang et al., 2012). Second, genetic manipulation of DIM-7, a nuclear transporter of pMAPK, regulates LTM consolidation bi-directionally in MB (Li et al., 2016). To clearly confirm this suggestion, *in vivo* imaging study will be helpful.

## Electronic supplementary material

Below is the link to the electronic supplementary material.

Supplementary material 1 (PDF 461 kb)
