# Supplementary material for: Preferential distribution of nuclear MAPK signal in α/β core neurons during long-term memory consolidation in Drosophila
Source: Protein Cell. 2017 Apr 19;8(10):780–3. doi: 10.1007/s13238-017-0404-8 (PMC5636746; doi:10.1007/s13238-017-0404-8)
Supplement: Supplementary file 1 — Supplementary material 1 (PDF 461 kb) [file 13238_2017_404_MOESM1_ESM.pdf]

Supplemental materials

Materials and Methods

Fly Stocks. Flies were raised at 25 ℃ with 60% humidity on standard cornmeal medium in 12/12h light/dark cycle. *VT44966, VT57244 and VT26665* were kindly provided by Dr. Ann-Shyn Chiang. *NP7175* was obtained from Dr. Kei Ito. *MB-247-dsRed* was acquired from Dr. André Fiala. The following stocks were obtained from the Bloomington Stock Center: *c739, UAS-mCD8::GFP* and *UAS-nlsGFP*.

Pavlovian Olfactory Aversive Conditioning. Flies were trained according to standard procedures as described previously (Li et al., 2016). Three- to five-day-old flies were used for conditioning in a dark room with 25 ℃ and 70% relative humidity. 3-Octanol (OCT) (Aldrich, 1.5×10^{-3} in dilution) and 4-methylcyclohexanol (MCH) (Fluka, 1×10^{-3} in dilution) were used as conditioned stimulus (CS), while 60V electric foot shock (twelve 1.5s pulses with 3.5s intervals) was used as unconditioned stimulus (US). In each single cycle training, about 100 flies were subjected to one CS accompanied by US (CS+), and then subjected to the other CS only (CS-). Spaced training (four cycles, 15 min interval) was used in massed training (no interval) and spaced training (15 min interval) to form long-term memory. This paradigm has been used and described previously. Flies after spaced training were kept at 18 ℃ to rest till dissecting.

Immunofluorescence. The procedures are also described previously (Li et al., 2016). Flies were quickly anesthetized on ice and dissected in ice-cold PBS within 5 minutes. Brains were fixed in 4% paraformaldehyde in PBS for 30 min on ice. All solutions added after fixation in pMAPK staining were treated with 1% phosphatase inhibitor cocktail (Thermo Fisher Scientific). After being washed three times in PBS, brains were treated in blocking solution (PBS with 2% Triton X-100 and 10% goat serum) for 60 min at room temperature. Then the brains were transferred into primary antibody solution (PBS with primary antibody, 0.2% Triton X-100 and 1% goat serum) and incubated for at least 24 hours at 4 ℃. Rabbit anti-pMAPK antibody (1:50, Cell Signaling Technology), mouse anti-nc82 antibody (1:10, DSHB), rabbit anti-DsRed (1:400, CloneTech) or chicken anti-GFP antibody (1:2000, Abcam) was used as primary antibody depending on the experiment. Brains were washed three times again in PBS and transferred into secondary antibody solution (PBS with secondary antibody, 0.2% Triton X-100 and 1% goat serum) and incubated overnight at 4 ℃. Goat anti-rabbit IgG Cy3 antibody (1:200, Jackson ImmunoResearch), goat anti-mouse IgG Cy3 antibody (1:200, Jackson ImmunoResearch) or goat anti-chicken IgG Alexa Fluor 488 antibody (1:200, Invitrogen) was used as secondary antibody depending on the experiment. Then TO-PRO-3 iodide in diluent buffer (1:500) was used to stain nuclei by incubating for 30 min at room temperature and finally washing with PBS three times. Brains were mounted in VECTASHIELD mounting medium (Vector Laboratories). Images were taken using confocal microscopy (Zeiss LSM710META).

Quantification of images. All images used for quantifications were carefully acquired avoiding overexposure or underexposure. For each genotype, data from flies were acquired at the same time and under the same microscope parameters. Four consecutive slices of each fly, which contained dendritic region of mushroom body (calyx) surrounded by Kenyon cells, were selected and calculated using Imaris software. Mean intensity of pMAPK in nuclei was divided by that in calyx to get the mean intensity ratio. For ratio of nuclear number, two consecutive slices of each fly, which contained dendritic region of mushroom body

(calyx) surrounded by Kenyon cells, were selected and calculated. All statistics were calculated from at least six brains.

Statistics. All data were analyzed using Graphpad Prism 6.0 software. Comparisons between two groups used two-tailed t-test. Comparisons of multiple groups used one-way ANOVA followed by Bonferroni *post hoc* comparisons. Statistically significance was showed with *, if P value < 0.05. All data in bar graphs are showed as means ±SEM.

Supplemental figures

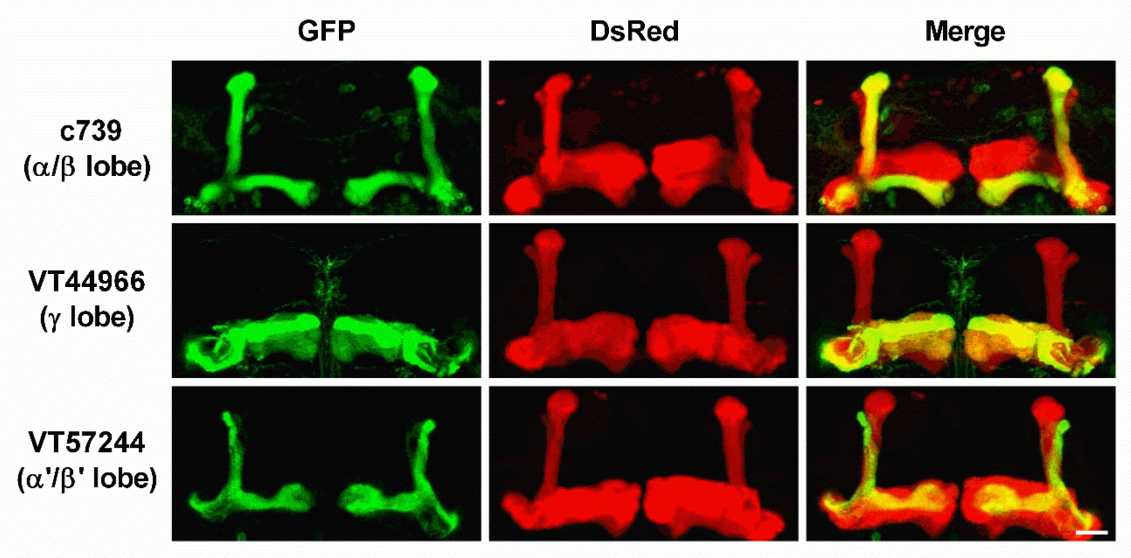

Fig. S1 Expression pattern of three different MB Gal4 lines. Indicated Gal4 lines were crossed to flies with the genotype *UAS-mCD8::GFP; MB247-DsRed* and identified by confocal imaging of whole adult central brain. Gal4 expression is displayed by green color. Structure of MB lobes is shown by red color. c739-Gal4, VT44966-Gal4 and VT57244-Gal4 showed strong expression in α/β lobe, γ lobe and α'/β' lobe respectively. Scale bar is 20 μm.

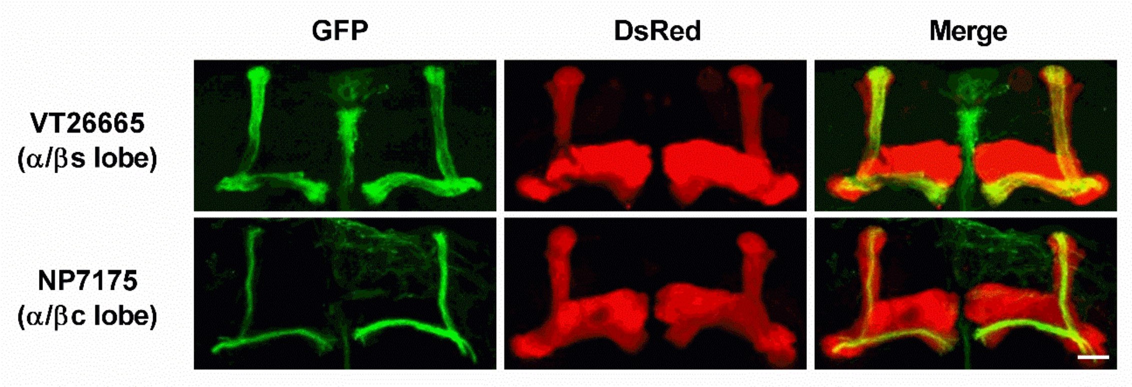

Fig. S2 Expression pattern of two different Gal4 lines of α/β KCs. Indicated Gal4 lines were crossed to flies with the genotype *UAS-mCD8::GFP; MB247-DsRed* and identified by confocal imaging of whole adult central brain. Gal4 expression is displayed by green color. Structure of MB lobes is shown by red color. VT26665-Gal4 and NP7175-Gal4 showed strong expression in α/βs lobe and α/βc lobe respectively. Scale bar is 20 μm.
